# Mechanical regulation of nucleocytoplasmic translocation in mesenchymal stem cells: characterization and methods for investigation

**DOI:** 10.1007/s12551-019-00594-3

**Published:** 2019-10-18

**Authors:** Lucia Boeri, Diego Albani, Manuela Teresa Raimondi, Emanuela Jacchetti

**Affiliations:** 1 Department of Chemistry, Materials and Chemical Engineering “Giulio Natta”, Politecnico di Milano, Piazza Leonardo da Vinci 32, 20123 Milan, Italy; 2 Department of Neuroscience, IRCCS - Istituto di Ricerche Farmacologiche Mario Negri, Milan, Italy

**Keywords:** Mechanotransduction, Mesenchymal stem cell, Nucleocytoplasmic translocation, Nuclear pore complex, Fluorescence microscopy

## Abstract

Mesenchymal stem cells (MSCs) have immune-modulatory and tissue-regenerative properties that make them a suitable and promising tool for cell-based therapy application. Since the bio-chemo-mechanical environment influences MSC fate and behavior, the understanding of the mechanosensors involved in the transduction of mechanical inputs into chemical signals could be pivotal. In this context, the nuclear pore complex is a molecular machinery that is believed to have a key role in force transmission and in nucleocytoplasmic shuttling regulation. To fully understand the nuclear pore complex role and the nucleocytoplasmic transport dynamics, recent advancements in fluorescence microscopy provided the possibility to study passive and facilitated nuclear transports also in mechanically stimulated cell culture conditions. Here, we review the current available methods for the investigation of nucleocytoplasmic shuttling, including photo-perturbation-based approaches, fluorescence correlation spectroscopy, and single-particle tracking techniques. For each method, we analyze the advantages, disadvantages, and technical limitations. Finally, we summarize the recent knowledge on mechanical regulation of nucleocytoplasmic translocation in MSC, the relevant progresses made so far, and the future perspectives in the field.

## Introduction

**Fig. 1:**
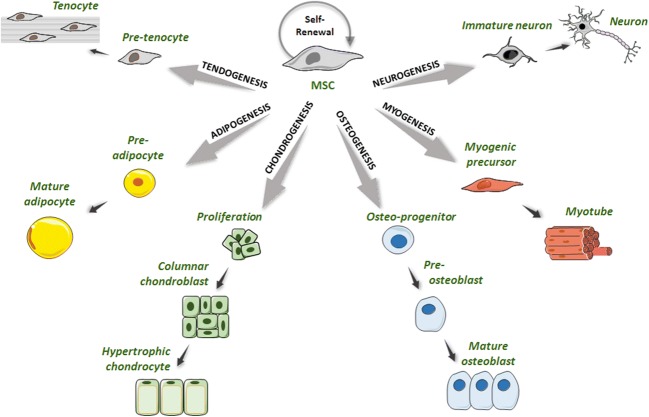
Graphical representation of mesenchymal stem cells differentiation pathways

Over 10 years, thanks to their unique properties and multiple clinical benefits, mesenchymal stem cells (MSCs) have been studied and used as suitable and promising tools for cell-based therapy applications. MSCs are adult multipotent cells with the great potential to self-renew and to differentiate into multiple cell lineages mainly derived from the mesodermal layer but also from the ectoderma and endoderma under specific conditions (Fig. 1) (Moon et al. 2018; Le and Yao 2017).

MSCs can be easily harvested from different mesenchymal tissues that are bone marrow, adipose tissue, umbilical cord, and dermis. Cell isolation is a simple procedure based on the collection of a heterogeneous population of plastic-adherent cells screened by the expression of specific surface antigens (Barry and Murphy 2004). The use of this type of adult stem cells arises low ethical issues (Volarevic et al. 2018), shows a low risk of tumorigenicity (Meier et al. 2013), and possesses a broad spectrum of immune regulatory and tissue organ repairing ability. Thanks to MSC immune-modulatory properties, these cells were used for some preclinical cell-based treatment of different autoimmune diseases, such as systemic lupus erythematosus (Cras et al. 2015), inflammatory bowel’s disease (He et al. 2012), and rheumatoid arthritis (Abd Elhalem et al. 2018). These first studies showed great benefits on clinical and biochemical markers but appeared highly dependent on the host inflammatory state. Exploiting their tissue-regenerative properties, MSCs have been also applied for cell-based therapy in case of several other pathologies, such as liver disease (Zhao et al. 2018), myocardial infarction (Madigan and Atoui 2018), pancreatitis (Ahmed et al. 2018), and stroke (Bang et al. 2016).

Focusing on improving the clinical cell application and the control of MSC fate and behavior, researchers have been able to modulate MSC differentiation by regulating the chemical or mechanical environment. Until some decades ago, they guided MSC differentiation by adding exogenous chemical molecules to cell culture. However, these techniques do not allow translation to the clinic because they could induce immune reactions or even cause tumors onset. The main challenge today is being able to effectively control the differentiation in vitro without using chemical factors. Therefore, with the aim of mimicking the entire bio-chemo-mechanical environment, recent promising approaches are focused on the mechanical influence of the MSC environment (Raimondi et al. 2012).

## Mechanical regulation of MSC differentiation

In the last decades, researchers evaluated how mechanical environment (matrix and external inputs) is transduced into a cell biochemical signal leading to gene transcription. This mechanism is defined as mechanotranscription. Among all the transcription factors involved in MSC differentiation pathways, few factors have been studied in the context of mechanotranscription. In Table 1, we listed the main transcription factors (TFs) involved in the promotion of the earlier stages of MSC differentiation. As this table clearly shows, so far, the pathways investigated in connection to the mechanotranscription mechanism are those characterizing the three mesodermal cell lineages defined by the International Society of Cellular Therapy for the determination of MSC population: adipogenesis, osteogenesis, and chondrogenesis.

**Table 1:** Characteristics of the main transcription factors (TFs) promoting the earlier stages of MSC differentiation. In italics, the TFs found to be involved in the mechanotranscription mechanism are highlighted

Differentiation pathways	Factors	Type	Molecular weight (kDa)	Ref.
Osteogenesis	*Runx2 (Cbfa1)*	Runt-related TF	18.8	Yanagisawa et al. 2007 Yang et al. 2014 Murphy et al. 2012 Hime and Abud 2013
	*Osterix*	Zinc Finger TF	44.9	Yanagisawa et al. 2007 Hime and Abud 2013
	*Dlx Family*	Homeobox TF	Dlx3 = 31.7 Dlx5 = 31.5 Dlx6 = 32.5	Yanagisawa et al. 2007 Hime and Abud 2013
Adipogenesis	*PPARγ*	Nuclear Receptor	54.7	Yanagisawa et al. 2007 Yang et al. 2014 Li et al. 2015 Case et al. 2013 Hime and Abud 2013
	*C/EBPS*	*Basic Leucine Zipper Domain TF*	α = 35.9 β = 36.1 γ = 28.4	Li et al. 2015 Hime and Abud 2013
	SREBP1/ADD1	Sterol regulatory element-binding TF	~ 49	Hime and Abud 2013
Chondrogenesis	*Sox9*	SRY-related High mobility group-box TF	56.1	Yanagisawa et al. 2007 Murphy et al. 2012 Hime and Abud 2013
	*Sox5*		84	Yanagisawa et al. 2007 Hime and Abud 2013
	Sox6		91.9	Yanagisawa et al. 2007 Hime and Abud 2013
	*Runx2 (Cbfa-1)*	Runt-related TF	18.8	Yanagisawa et al. 2007 Yang et al. 2014 Murphy et al. 2012 Hime and Abud 2013
Myogenesis	*Myod*	Basic helix loop helix TF	34.5	Yanagisawa et al. 2007 Pownall et al. 2002
	Myogenin		25	Pownall et al. 2002
	Myf5		28.3	Pownall et al. 2002
	Myf6 (MRF4)		26.9	Pownall et al. 2002
Tenogenesis	Scleraxis	Basic helix loop helix TF	21.6	Wang et al. 2018
	Mohawk	Homeobox TF	39.4	Liu et al. 2015
	Egr1	C_2_H_2_-type zinc finger TF	57.5	Guerquin et al. 2013
Neurogenesis	Ascl1	Basic helix loop helix TF	25.5	Araújo et al. 2018
	Neurogenin		25.7	Araújo et al. 2018 Schäck et al. 2016
	Foxa2	Forkhead box transcription protein	48.9	Marrelli et al. 2015

**Fig. 2:**
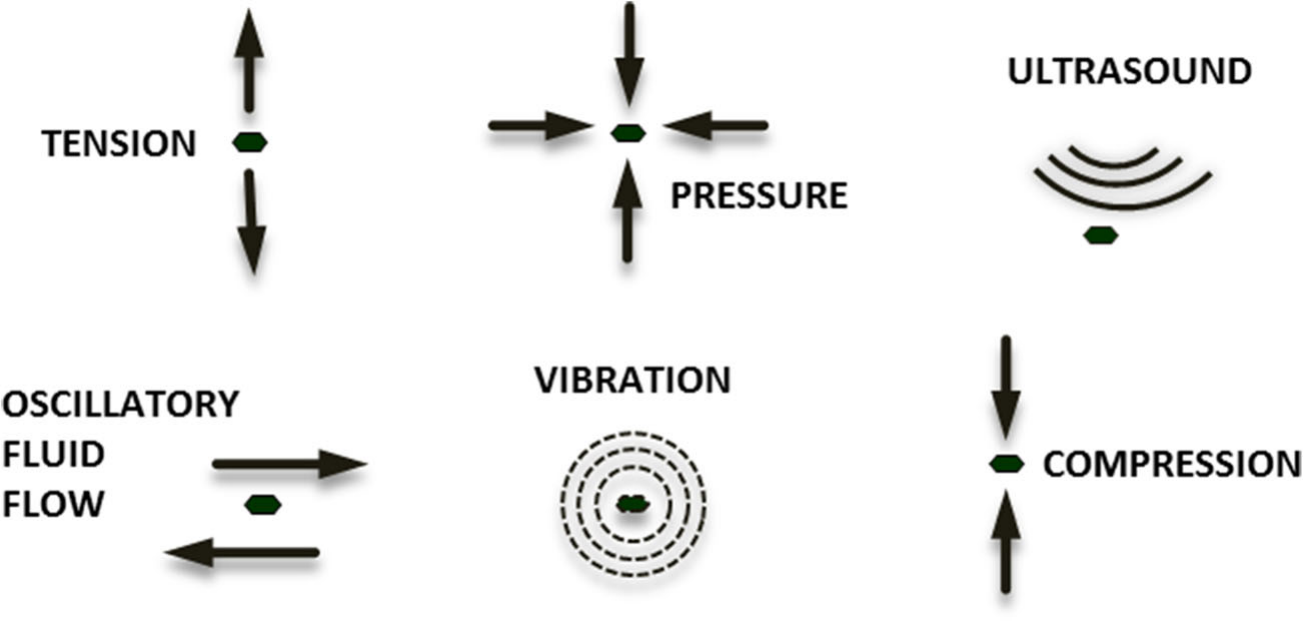
Mechanical stimuli characterizing physiological MSC environment: hydrostatic pressure, tensile stress, fluid flow, compression, vibration, ultrasound

Matrix microenvironment (architecture, stiffness, composition) and external mechanical stimuli clearly influence both in vitro and in vivo cell growth. Substrate and matrix stiffness regulate cell properties, such as differentiation, proliferation, and cell shape (Nava et al. 2012, 2014; Sun et al. 2018). For example, soft substrates, mechanically similar to the brain tissue, were found to stimulate neurogenesis; myogenesis instead was induced by an intermediate substrate stiffness; relatively stiff substrates were finally shown to promote osteogenesis (Guilak et al. 2009; Engler et al. 2006). In the same way, external mechanical stimuli, reproducing the physiological mechanical condition characteristic of the MSC niche, cause biological and structural cell rearrangements. The main external mechanical cues are hydrostatic pressure, tensile stress, fluid flow, compression, vibration, and ultrasound (Fig. 2). Below we list the main effects on MSC differentiation applying each mechanical stimulus.

Hydrostatic pressure (HP) is a non-deforming mechanical stimulus able to increase chondrogenic gene expression in MSCs (Luo and Seedhom 2007). HP promotes mechanotransduction altering ions concentration, such as Na^+^ and Ca^2+^ (Wright et al. 1992; Browning et al. 1999), and cytoskeleton organization, involving both microtubules and vimentin rearrangement (Jortikka et al. 2000; Steward et al. 2013).

Tension loading is an external mechanical force able to stimulate MSCs to tenogenic, osteogenic, or myogenic differentiation. Depending on the strain intensity, MSCs show a specific differentiation fate (Park et al. 2004; Chen et al. 2008). For example, Chen and colleagues subjected human bone marrow MSCs to 3% and 10% strain observing osteogenesis and tenogenesis, respectively (Chen et al. 2008).

Oscillatory fluid flow (OFF) induces shearing stress and it is found to promote both osteogenic than myogenic differentiation. The method commonly used to induce this type of stress involves perfusion bioreactors with a steady, pulsatile, and unidirectional flow (Sikavitsas et al. 2003). Fluid flow has consistently been demonstrated to promote osteogenesis and myogenesis in bone marrow MSCs (Huang et al. 2010).

Compression loading strongly promotes chondrogenic differentiation of MSCs upregulating chondrogenic markers gene expression, such as collagen II and aggrecan, without any exogenous factors stimulation (Huang et al. 2004). A smaller number of studies also demonstrated that this type of external mechanical signals can induce MSC osteogenic differentiation increasing bone matrix formation and calcium deposition (Sittichokechaiwut et al. 2010).

Finally, vibration has been found to promote osteogenesis, increasing the expression of osteogenic markers, such as osteopontin and osteocalcin (Sen et al. 2011; Yourek et al. 2010), and low-intensity-pulsed ultrasound (LIPUS) has been shown to direct chondrogenic differentiation of rat MSCs promoting the matrix formation and increasing the expression of chondrogenic markers, such as COL2A1 and Sox-9 (Lee et al. 2006).

## Cell and nuclear mechanosensors

**Fig. 3:**
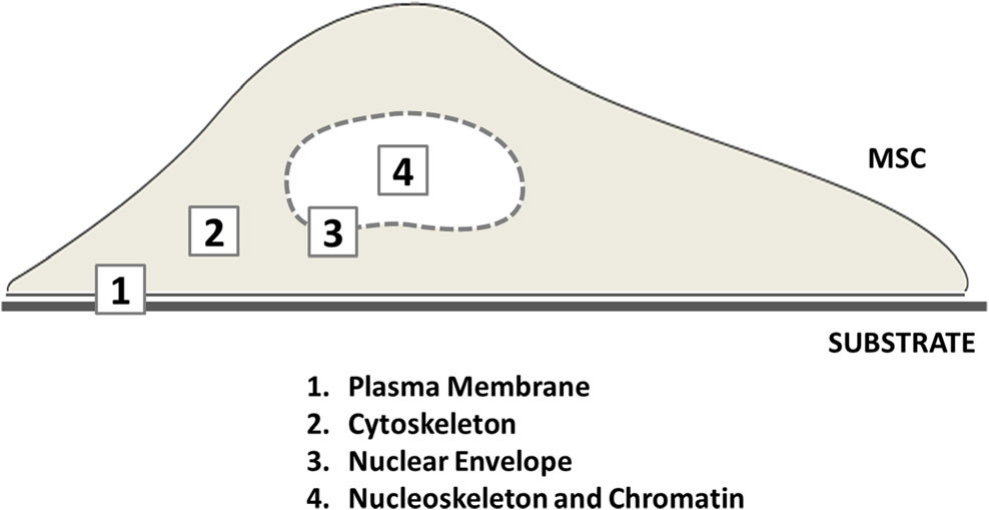
Sketch representing how MSCs respond to mechanical stimuli and to substrate rigidity with several biological mechanosensors distributed throughout the cell. Sequentially, plasma membrane components (1) capture the external mechanical signal via integrins, cadherins, and focal adhesions, and transmit it to cytoskeletal elements (2) that propagate the input throughout the cell until the nucleus. The nuclear envelope structural elements (3) can transduce the signal to the lamin A/C and the chromatin (4) modulating the accessibility of transcription factors to DNA and thus the transduction of the mechanical stimulus into chemical signals

From a molecular point of view, the mechanical mechanisms that trigger or influence cell structure rearrangements leading to biochemical signals are still not fully understood. In this context, the players involved in the mechanotransduction event include several elements: proteins of the plasma membrane and cytoskeleton, nuclear complexes and structures, and DNA (Fig. 3) (Bonnet and Ferrari 2010).

The extracellular matrix (ECM) is the first element acting in the mechanotransduction mechanism. Some cell membrane elements capture chemical and mechanical signals from the extracellular environment and transmit them through the cell to the nucleus. These membrane components are integrins, cadherins, cilia, and ion channels. Integrins bind cytoskeleton through focal adhesion complexes and functioning as the direct linkage between ECM and intracellular environment. Thus, thanks to integrins, external stimuli could be transmitted and translated into a structural rearrangement of cytoskeleton organization. Other sensors of external inputs are ion channels, Piezo proteins, which respond to stimuli altering the intracellular cationic flow and triggering intracellular mechanisms (Wu et al. 2017; Chubinskiy-Nadezhdin et al. 2017). For instance, cadherins that are cell-cell junction proteins are involved in mechanotransduction, thanks to their dependence to calcium concentration. In fact, in response to its variation, cadherins change their molecular conformation leading to cytoskeleton rearrangement and the promotion of signaling molecule release (Arnsdorf et al. 2009). Calcium concentration indirectly modulates the actin dynamics also via Rho-A/Rock promotion (Haws et al. 2016).

The actin cytoskeleton is the second main player in the force transmission: cell adhesion induces the formation of focal adhesions that are the protein complexes, able to support the formation of long and strong actin filaments. The larger the focal adhesions are, the more the cell will be able to transmit internal forces to the nucleus, through actin bundles.

In the force transmission pathway, the third group of players is the LINC complex, the nuclear pore complex (NPC), and the lamina. As focal adhesions connect ECM to the cytoskeleton, LINC (LInker of Nucleoskeleton and Cytoskeleton) complexes connect the cytoskeleton to the nucleoskeleton, leading to the transmission of an external stimulus to the internal environment of the nucleus (Lombardi et al. 2011). Among the nucleoskeleton elements, lamin A\C is the component more involved in nuclear structure stabilization against mechanical stress (Swift et al. 2013).

Together with the LINC complexes, the nuclear pore complex (NPC) is the other structural linkage between the cytoplasm and the nucleus. Recently, researchers have shown that LINC complexes and NPC are directly connected (Swift et al. 2013). Unfortunately, the NPC real involvement in mechanotransduction is still not fully understood and it remains a challenge. Globally, the transmission of the mechanical loading by LINC complex and NPC to the nucleocytoskeleton alters the lamin A\C structure and organization. Since lamina strictly interacts with chromatin (Oldenburg and Collas 2016), its skeleton destabilization is transduced in chromatin rearrangement and epigenetic modifications, leading to the exposure of specific binding sites to the transcription machinery (Killaars et al. 2018; Heo et al. 2015; Arnsdorf et al. 2010). Thus, besides the chromatin remodeling, understanding the nucleocytoplasmic shuttling of proteins—such as transcription factors—could be determined to fully characterize and control the mechanotransduction events.

In a famous study, Sirio Dupont highlighted an interesting phenomenon related to the migration of transcription factors involved in key cellular mechanisms. He showed how two mechanotransduction mediators YAP (yes-associated protein)/TAZ (transcriptional coactivator with PDZ-binding motif) localization is strictly related to the rigidity and cell shape (Dupont et al. 2011). They plated MSCs on micropillars arrays of different rigidity and observed a higher percentage of nuclear YAP/TAZ when MSCs were plated on rigid micropillars with rather than elastic ones.

Within this scenario, the aim of this review was to summarize the state of the art of the nucleocytoplasmic transport through the nuclear pore complexes in mesenchymal stem cells subjected to mechanically stressed conditions.

## The nuclear pore complex

**Fig. 4:**
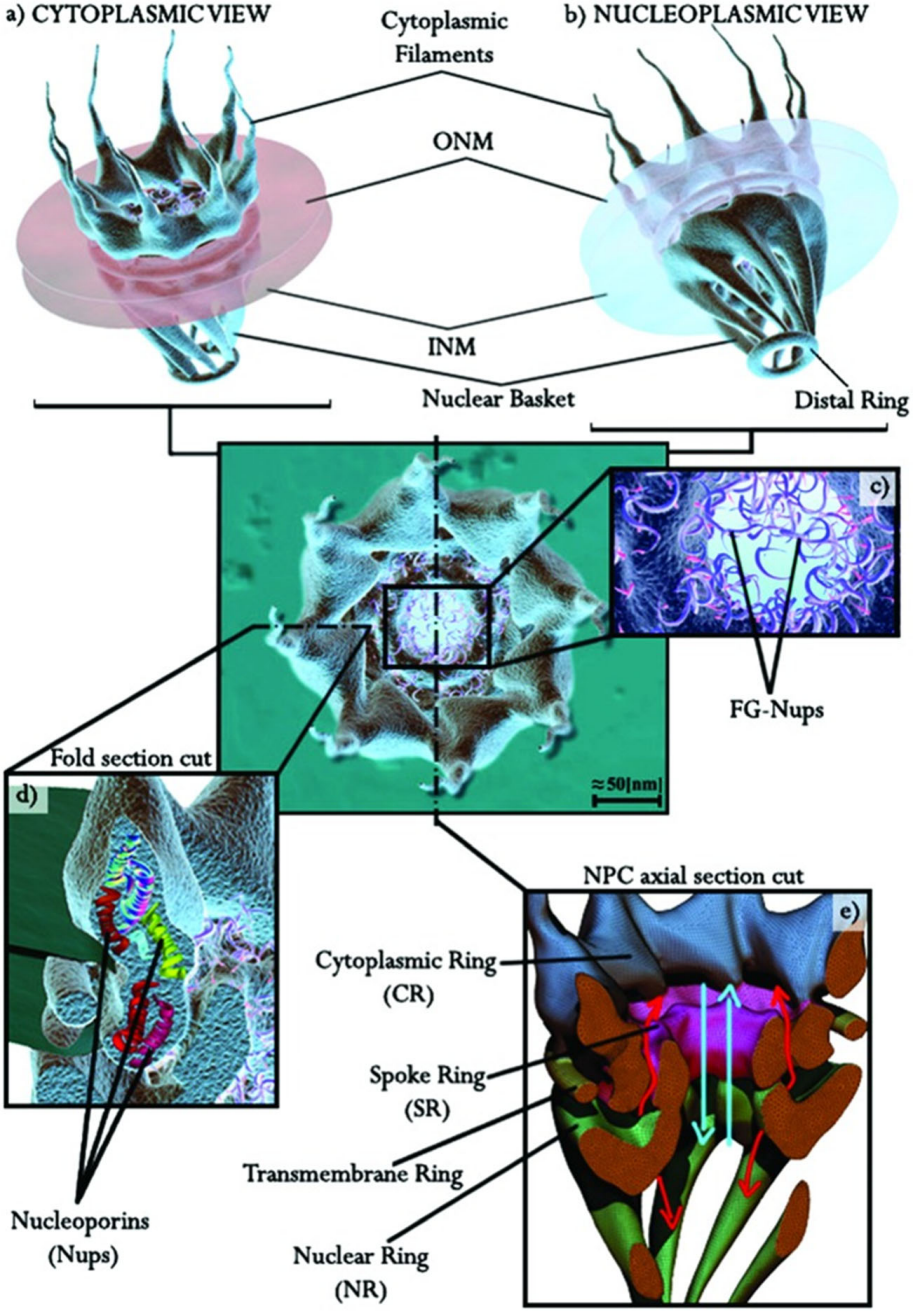
Scheme of the nuclear pore complex. (**a**) Cytoplasmic and (**b**) nucleoplasmic view of the NPC. (**c**) Illustration of FG-nucleoporin filament in the NPC central channel. (**d**) Illustration of nucleoporins (Nups) composing a section of the NPC. (**e**) Vertical section of the NPC: the cytoplasmic ring (CR) is marked in blue, the central ring/spoke ring (SR) is shown in purple, the nucleoplasmic ring (NR) and the nuclear basket filaments are labelled in green. The translocation paths are represented with light blue arrows and the red ones are representative of the secondary channels. Image taken from (Garcia et al. 2016) with permission

The NPC is a complex molecular machine (∼ 125 MDa) composed by approximately 30 nucleoporins (Nup), giving to the NPC a cylindrical structure that spans from the cytoplasmic to the nucleoplasmic side of the cell (Fig. 4) (Garcia et al. 2016). In the cytoplasm side, the gate is the cytoplasmic ring: it is a diaphragm with a diameter around 100–150 nm equipped by eight filaments (50–70 nm in length) catching cargoes and macromolecules to facilitate their transport to the nucleus.

The spoke ring is the NPC structure localized in the nuclear envelope (NE) lipid bilayer. It is a cylinder of 50-nm length and width between 20 and 80 nm, filled with protein filaments, and the phenylalanine–glycine repeats nucleoporins (FG-Nups). FG-Nups have the role to block big inert molecules (> 70 kDa) and facilitate cargo passage to the nucleus. The exchange of ion and small molecule is also allowed from a group of secondary channels (around 4 nm in diameter), placed around the spoke ring. The transmembrane ring located between the two NE lipid membranes confers stability to the NPC cylinder.

Finally, the nuclear and the distal rings are in the nucleoplasmic side and are connected by Nup153 and Tpr proteins forming the nuclear basket (50–75-nm length) (Gu 2018; Garcia et al. 2016; Wente and Rout 2010).

Transports through the NPC could be passive or facilitated. They are bidirectional and share the central diffusion channel located within the central pore: molecules smaller than ~ 70 kDa in size (corresponding to a maximum diameter ~ 10 nm) can passively diffuse across the central part of the pore and their translocation capability is function of size (Gerace and Burke 1998; Keminer and Peters 1999; Paine et al. 1975).

Molecules bigger than 70 kDa pass through the NPC by facilitated diffusion only in the presence of specific motifs (nuclear localization and nuclear export signals (NLS/NES)). The carrier is aided to translocate principally by FG-Nup filaments with cell energy expenditure. In fact, the release of molecules into the nucleus or into the cytoplasm is driven by the state of the Ran nucleotide that cycles between the GDP and the GTP bound states.

NPC molecular machinery is so efficient that in a single pore could translocate up to 1000 molecules/s corresponding to a mass flow nearly 100 MDa/s (Ribbeck and Gorlich 2001; Stewart 2007). In the last decades, the development of genetically encoded fluorescent proteins and fluorescent synthetic dyes has opened the door to study protein localization and trafficking at the level of single cell and single pore (Chalfie 1995; Los et al. 2008; Keppler et al. 2002; Giepmans et al. 2006). Different translocation models have been proposed and well described (Fahrenkrog and Aebi et al. 2003). Nevertheless, since the mechanism of nucleocytoplasmic translocation remains poorly understood, it is currently intensely investigated with the techniques described in the following section.

## Fluorescence microscopy techniques applied to characterize nucleocytoplasmic transport

**Fig. 5:**
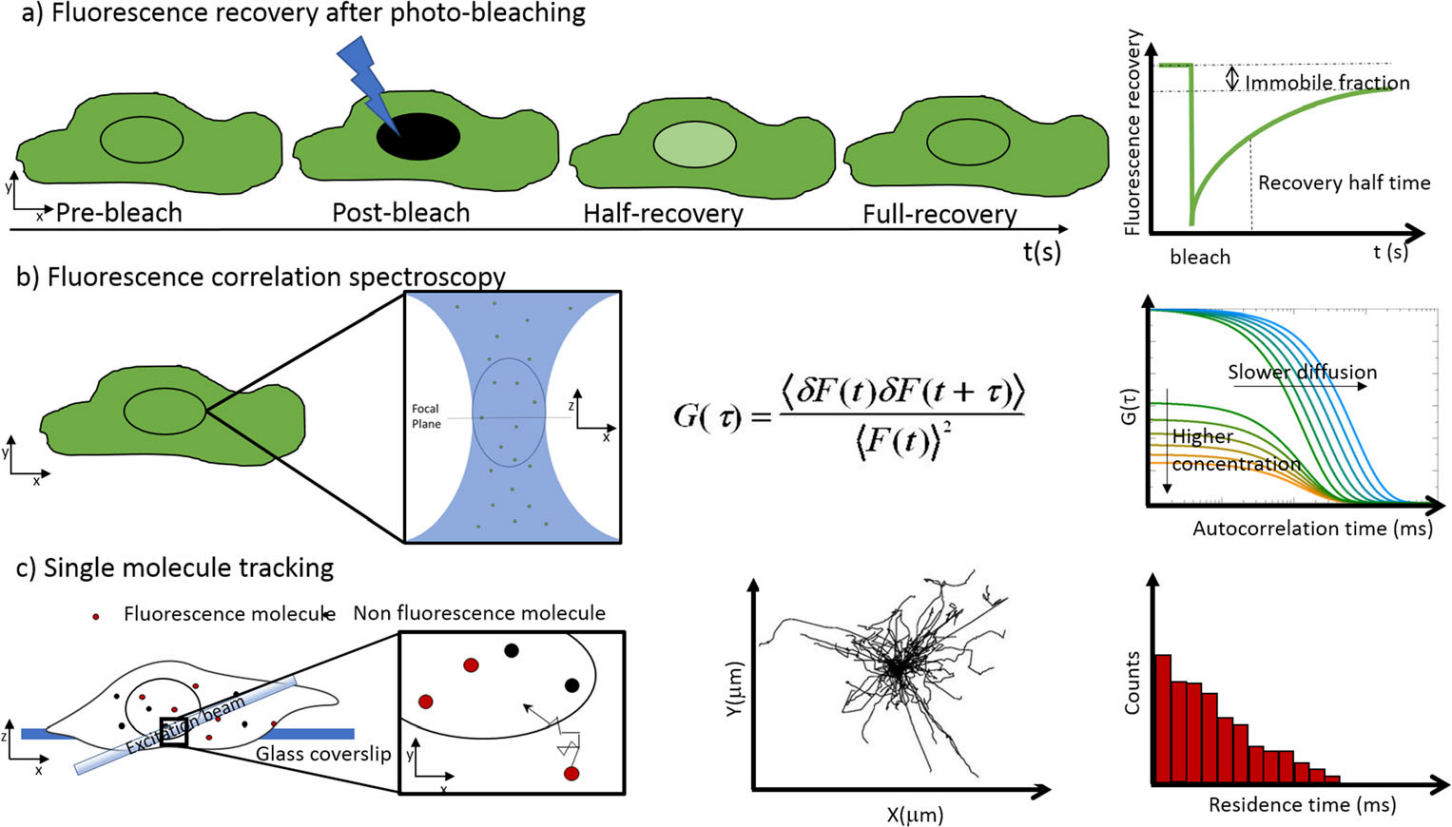
Schematic representation of three approaches to measure molecule nucleocytoplasmic translocation. **a** FRAP is the most used photo-perturbation technique. It is based on the nuclear photo-bleaching and the subsequent measure of the fluorescence recovery as intensity average of the cell nucleus. Fast recovery is indicative of diffusion and a strong molecule binding leads to an immobile fraction of non-fluorescent proteins. **b** FCS is a fluctuation-based method. It measures fluorescence fluctuations arising from fluorescent proteins movement across the excitation volume. The autocorrelation function *G*(*τ*) represents the probability that the protein of interest remains within the excitation volume for a time longer than *τ*. **c** SMT is based on the detection of individual fluorescent proteins. This is the reason why it requires a very low concentration (pM) of fluorescent molecules. To maximize the signal to noise ratio, the excitation occurs by tilting the laser beam (HILO illumination)

Fluorescence microscopy provides an efficient approach to study diffusion and transport in and out subcellular compartments. In fact, in the last decade, significant advances have been made not only in the field of fluorescent dye\protein engineering but also in microscope set-up development and quantitative fluorescence microscopy techniques. Today, molecular events can be studied both at the level of the single live cell (microns) and single pore (nanometers), allowing visualization and analysis of molecular dynamics through a single NPC. The main techniques used to evaluate fluorescent molecule and protein diffusion or fluxes between different cell compartments are photo-perturbation, correlation spectroscopy, and single-molecule tracking (Fig. 5 and Table 2).

**Table 2:** Features summary regarding the techniques for nucleocytoplasmic transport investigation

Techniques	Spatial resolution	Temporal resolution	Sample preparation	Labelling technique	Molecule concentration	Extracted information
Photo-perturbation	Microscale	Seconds	Transfection, microinjection	Fluorescent proteins	Micromolar	Molecule ensemble
Correlation spectroscopy	Nano/microscale	Milliseconds	Transfection, microinjection	Fluorescent proteins	Nanomolar	Molecule ensemble
Single-molecule tracking	Nanoscale	Milliseconds	Transfection, microinjection, permeabilization	Organic dye or quantum dot	Picomolar	Single molecule

Photo-perturbation approaches are based on changing the fluorescent dye photo-physical properties and studying the redistribution of fluorescence. Among these techniques, Fluorescence Recovery After Photo-bleaching (FRAP) is a powerful method used to investigate protein mobility into the cells (Reits and Neefjes 2001; Kang et al. 2009). It consists of irreversibly photo-bleach fluorescent protein in a selected region of interest (ROI). Thanks to the protein diffusion, it is possible to analyze the fluorescence recovery into the ROI. Two main parameters can be extracted from the FRAP measurement: the mobile fraction (*M*_f_), representing the protein fraction diffusing in the selected ROI, and the characteristic time of the diffusion *t*_1/2_. Small and highly diffusing proteins show fast recovery, while no fluorescence recovery is observed with an immobile molecule. The speed of the recovery is dependent on the molecular size, the environment viscosity, or the interaction degree between the protein of interest and other molecules (Lippincott-Schwartz et al. 2001).

In photo-perturbation experiments, the protein of interest is linked to a fluorescent protein or dye chosen relying on their physico-chemical features such as quantum yield, its low tendency to photo-bleach, and its photo-stability during post-bleach image acquisition (Lippincott-Schwartz and Patterson 2003). FRAP measurement is widely used and is relatively easy to perform. Furthermore, it can be accomplished on any standard confocal microscope or on a wide-fields microscope equipped with a laser able to bleach a limited area. In any case, it is necessary to keep in account that this technique has some disadvantages (Bancaud et al. 2010; Mueller et al. 2012, 2013; Mazza et al. 2007; Braeckmans et al. 2003; Blumenthal et al. 2015):

- The high expression level of fluorescence is required, precluding the study of proteins that need to be over-expressed.
- Photo-bleaching is generated by a strong laser pulse that could induce cell photo-toxicity.
- Photo-bleaching is not completely irreversible for several fluorescent molecules based on GFP technology, leaving some uncertainty on the measure of goodness.
- Results are dependent on the size of the bleached ROI and the profile of the bleached volume making it difficult to measure diffusion on large areas and volumes.
- A quantitative interpretation of FRAP measurements is not trivial and data need to be fitted with the correct kinetic model.

Another photo-perturbation method more recently used is the fluorescence loss in photo-bleaching (FLIP). It could be used in place or paired with FRAP to investigate proteins diffusion. This technique is based on the cell image acquisition between bleaching pulses on a fixed ROI. In this case, if the protein is free to move and able to enter in the bleaching region, a fluorescent signal decay is measured over the entire cell (Ishikawa-Ankerhold et al. 2012). Among photo-perturbation approaches, there are also emerging techniques based on photo-switchable and photo-activatable proteins. After an UV laser pulse, these molecules can shift their emission spectrum or switch from a dark state to a bright state (or vice versa) allowing to study protein mobility. Since the optical properties modulation can be induced with relatively low laser intensities, with these tools, it is possible to perform diffusion measurement, limiting the sample photo-damage (Bancaud et al. 2010).

First investigations to study passive diffusion process through the NPC in single live cells started using inert fluorescent proteins microinjected in *Xenopus ovocyte* or transfected in eukaryotic cells. In 2003, Wei et al. (2003) used FRAP to prove that EGFP (an inert fluorescent protein of 27 kDa) diffuses bi-directionally through the pore with a rate reduction up to ∼ 100-fold with respect to the diffusion within the nucleus or the cytoplasm, due to the reduced size of the NPC channel available. Moreover, any significant variation in EGFP diffusion through the NPC was observed by Ca^2+^ depletion demonstrating that EGFP nucleocytoplasmic translocation is a passive diffusion event.

After the pioneering studies on inert tracers, photo-perturbation techniques were successfully used to examine the nucleocytoplasmic-facilitated transport of other molecules (Ando et al. 2004; Köster et al. 2005; Sunn et al. 2005; Chudakov et al. 2007; Davies et al. 2010; Cardarelli et al. 2011a), such as the proteins with nuclear localization/export signals (NLS/NES) and importins. Measures confirm the hypothesis that facilitated nuclear import regulation is mediated by the binding with the β-domain of importinα and that both passive and facilitated transports occur through the central pore channel without interfering each other. Otherwise, different molecules transported by the same pathway hamper each other (Naim et al. 2007; Cardarelli et al. 2009; Bizzarri et al. 2012).

In fluorescence correlation spectroscopy (FCS), a small volume of the sample is illuminated and specimen fluorescent fluctuations are acquired over time. Using the autocorrelation function G, fluorescence fluctuations provide information about molecules concentration and diffusion. In fact, G amplitude is inversely proportional to the average number of fluorescent molecules and the decay time represents the diffusion capability (Elson 2011). FCS is a relatively non-invasive technique and it works best at low molecule concentration (nM concentration with respect to the μM concentration for FRAP), which hinders the use of simple transient transfections to make cells fluorescent. In contrast to FRAP, FCS is well suited for fast diffusion systems (in the order of sub-millisecond) and it is able also to determine the density and aggregation state of the protein of interest.

Using photo-perturbation approaches or FCS, it is possible to measure the mobility properties corresponding to the average behavior of the observed molecules, but the data need to be fitted with the right diffusion model to avoid inaccurate interpretation (Mueller et al. 2012, 2013). The main limitation of FCS is to provide information on a single point of the sample that is often not useful in a non-homogeneous system–like cells. For these kinds of samples, new techniques were developed, including both spatial and temporal correlation of fluorescence that reveals the direction and velocity of systematic motion. For example, the pair correlation fluorescence (pCF) technique has been widely used to study nucleocytoplasmic protein translocation. Its basic principle is to measure the time it takes from a molecule to migrate between two points, analyzing fluorescence fluctuation on a linear ROI. The spatial and temporal correlation among two arbitrary points of the line provides a map of protein transport and shows the presence of barriers or obstacles to diffusion with a millisecond time resolution (Cardarelli and Gratton 2010). Thanks to these technical advancements, in the last decade, correlation spectroscopy techniques appeared as ideal alternative strategies to investigate nucleocytoplasmic shuttling (Cardarelli et al. 2011b). In particular, the pair correlation function (pCF) method guarantees single-molecule sensitivity also in samples with high concentration of fluorescent molecules. pCF is therefore suitable to investigate the nucleocytoplasmic translocation of fluorescent proteins and the role of the NLS in nuclear-facilitated transport. Cardarelli et al., for example, calculated the NLS-GFP transit time through the nuclear pore in the 1–40-ms range and demonstrated that fastest cytoplasm-to-nucleus transit happens very close to the NE barrier, where endogenous importin carriers are accumulated (Cardarelli and Gratton 2010).

The single-molecule tracking (SMT) technique allows to detect and track in time and space individual fluorescent particles. It provides rich data sets that describe diffusion and binding kinetics of the protein of interest (Liu et al. 2016). During an image acquisition, a fluorescent single molecule produces a spot limited by the diffraction law. If molecules are at a very low concentration, they can be resolved and localized with a precision up to 20 nm by using post-processing algorithms (Mortensen et al. 2010). The particle localization precision depends, besides the molecule concentration, on the signal to noise ratio (SNR) of the images. Therefore, to maximize localization and SNR, it is primary to minimize the contribution of out-of-focus molecules and to express protein at very low concentration (pM), for example by using microinjection techniques or new labelling technology (like SNAP-TAG or HALO-TAG approaches). To avoid imaging photo-bleaching it is suitable to use very stable and bright fluorophores (Chow et al. 2016; Los et al. 2008; Keppler et al. 2002). Therefore, to maximize the SNR, a highly illuminated and laminated optical light sheet (HILO) set-up is used: the highly inclined and thin laser beam creates an excitation volume in a limited depth range (microns) and in the center of the object field. Since only a thin layer of the sample is illuminated, the SNR increases about eight times with respect to epi-illumination (Tokunaga et al. 2008). Using this technique, the frame rate acquisition of the particle tracking spans from 100 to 10,000 frames/s, depending on the speed of the camera readout and the illumination time necessary to detect the optical probe (Liu et al. 2016). It allows a wide range of investigations like particle tracking, molecular interaction site, and molecule association/dissociation kinetics (Loffreda et al. 2017; Cui et al. 2018). Using the single-molecule tracking to analyze the nucleocytoplasmic transport allowed to show that the movement along the pore axis is bidirectional and it has the characteristics of a random walk. Furthermore, this technique provided a measurement of the interaction time (or residence time) between the fluorescent molecule and the pore, which is the spending time the protein takes to interact with the nuclear pore central channel proteins (Yang et al. 2004; Kubitscheck et al. 2005; Dange et al. 2008). The range spans from 1 to around 33 ms, as a function of the protein feature, but most of the proteins take 5–10 ms to cross the NPC (Tu and Musser 2011). Interestingly, not all signal-dependent nuclear import events complete the translocation through the NPC. In fact, molecules in proximity to the cytoplasmic periphery and those partially penetrated the central channel can more easily abort the transport, spending the majority of its interaction time moving within the NPC central pore. Moreover, cargo signal-dependent transport efficiency is a function of importin concentration. In fact, for example, NLS-2XGFP flux is reduced up to 50% in case of low importinβ level (Yang 2004; Yang and Musser 2006). Recently, by introducing the single-point edge-excitation sub-diffraction microscopy method (SPEED) again, Yang and colleague obtained a three-dimensional density map of the transient interactions with a spatiotemporal resolution of 9 nm and 400 μs (Ma and Yang 2010; Goryaynov et al. 2012; Goryaynov and Yang 2014). They demonstrated the following:

1. Electrostatic interaction between transiting molecules and FG-Nups does not play a dominant role in determining nuclear transport;
2. The spatial density of interaction sites between importinβ1 and FG-Nups increases as a function of the space and reaches its maximum in the central pore region;
3. Cargo rarely occupies the central NPC channel to pass from the cytosol to the nucleus;
4. The facilitated translocation pathway strictly depends on the FG-Nups interaction.

## Mechanoregulation of nucleocytoplasmic translocation

While it has been extensively proven that mechanotransduction regulates cellular mechanisms, such as cytoskeleton organization and gene regulation (Buxboim et al. 2010; McMurray et al. 2015; Bao et al. 2018; Keeling et al. 2017; Tajik et al. 2016; Miroshnikova et al. 2017), only in recent years a new idea is emerging that mechanical signals are transduced also by the nuclear membrane and, in particular, by the NPC. The connection between focal adhesions and the nucleus via the actin cytoskeleton likely allows to transmit internal forces that stretch the nuclear membrane and the NPCs, reducing the resistance to molecular transport through the nuclear membrane, thus increasing the molecules fluxes (Garcia et al. 2016).

The current understanding of mechanosensing at the nuclear envelope by NPC stretch activation and its possible effect in physiology and pathology is still poor (Donnaloja et al. 2019).

Currently, there are two theories concerning the mechanical opening of the pore. The first suggests that the tensions inside the cell stretch the nuclear envelope, increasing the pore size (Elosegui-Artola 2018). However, nowadays, the measurements supporting this theory are not completely reliable because they are carried out with a standard transmission electron microscopy procedure. This means that the sample must be fixed and included in resin, then cut using the microtome to obtain the slices imaged by the TEM. The use of a sliced plane does not allow to know the effective direction and depth of microstructures and, therefore, involves a systematic error in the measure. To overcome this limitation, it would be necessary to use a scanning EM tomography (STEM) or a focused ion beam combined with scanning electron microscopy (FIB-SEM) that allows a three-dimensional imaging of the sample and the measure of the effective dimension of the NPC without parallax errors. Using the STEM microscopy, we measured the nuclear ring area in non-adherent MSCs (which take roundish shape) and spread MSCs finding no significant differences (Garcia et al. 2016).

The second theory, which is yet to be proven, is that the cellular internal forces act on the nuclear part of the nuclear pore and precisely on the basket. The hypothesis seems to be reasonable, since it would give an explanation to the presence of the basket in the nuclear pore complex and above all plausible since the basket is formed by eight nucleoplasmic filaments resulting in a rotational symmetry (Lezon et al. 2009; Knockenhauer and Schwartz 2016). An external force, coming from the cytoskeleton and acting on the basket, could unroll the net facilitating the passage of TF collected in the basket (Donnaloja et al. 2019).

Apart from the opening mechanism of the pore that still must be investigated, what is known today is that in a cell subjected to mechanical stimulation, the transcription factors flow towards the nucleus increases. Elosegui-Artola and colleagues studied the NPC mechanotransduction evaluating the YAP nucleocytoplasmic translocation in fibroblasts (Elosegui-Artola 2018). YAP is a mechanosensitive transcription factor, notoriously involved in cancer, regeneration, and organ size control. They analyzed several elements related to the NPC mechanotransduction by using the FRAP technique. They demonstrated that applying forces to the cell nucleus, the YAP nuclear translocation increased by decreasing the restriction of NPC to protein transport. Moreover, modulating the stiffness of the substrate or using drugs to depolymerize the cell cytoskeleton, they have proven that YAP translocation was mediated by forces transmitted to the nucleus and that the import is related to the force transmission via actin cytoskeleton and not microtubules organization.

**Fig. 6:**
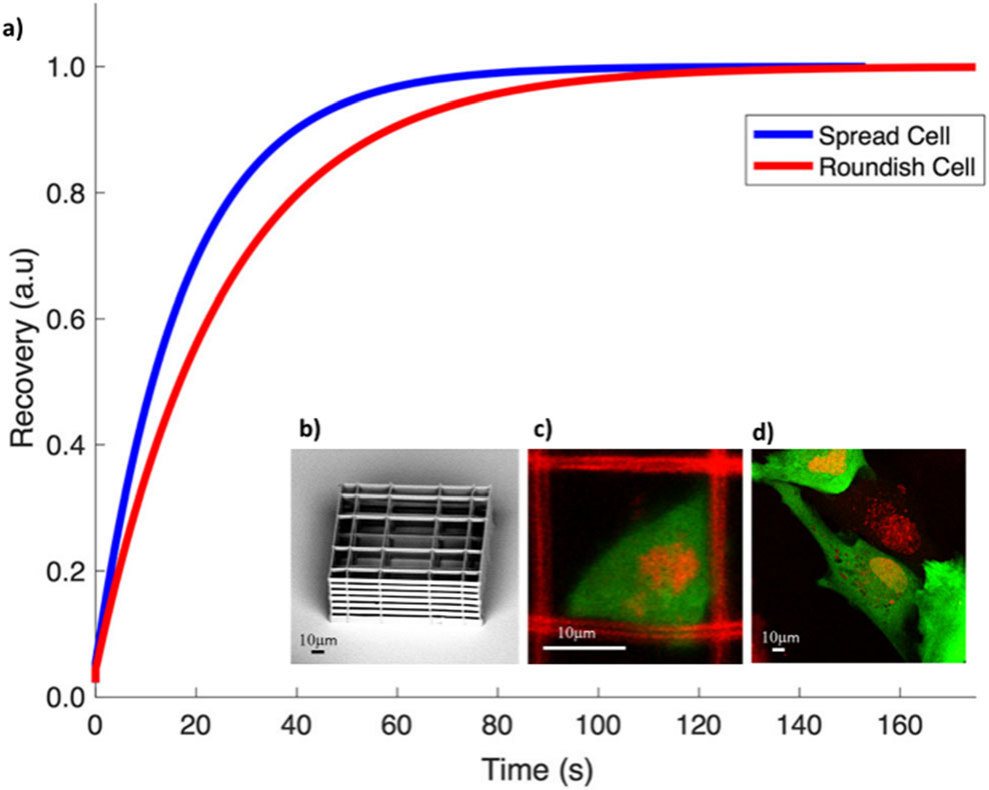
GFP fluorescence recovery after photo-bleaching curves measured on spread cells adhered on a flat substrate or roundish cells adhered in a Nichoid. (**b**) SEM image of a Nichoid. (**c**) Examples of GFP-expressing MSCs (green) grown into the Nichoid. In red, the Nichoid fluorescence and the cell DNA are visible. (**d**) Examples of GFP-expressing MSCs (green) grown on flat glass substrate. In red, the cell nuclei are highlighted

In the last years, we are working on the TF nucleocytoplasmic transport in MSCs. We evaluated the inert green-fluorescent protein (GFP) nucleocytoplasmic passive diffusion in MSCs grown on flat substrates or in three-dimensional substrates able to modify cell morphology (García-González et al. 2018). We cultured MSCs in a three-dimensional (3D) substrate, the “Nichoid,” able to condition cell adhesion at the single-cell scale, in order to maintain a roundish nuclear configuration, and on a flat glass substrate where the spread cell configuration induces a disk-like shape to the nucleus. We set up a numerical model of diffusive molecules transport through the NE, based on NPC deformation, and we compared results with those obtained measuring the GFP diffusion through the nuclear envelope by fluorescence recovery after photo-bleaching (FRAP). Our results show that cell stretching modulates the characteristic time needed for passive nuclear import of diffusive molecules, correlating a faster import with the nuclear spreading (Fig.6).

**Fig. 7:**
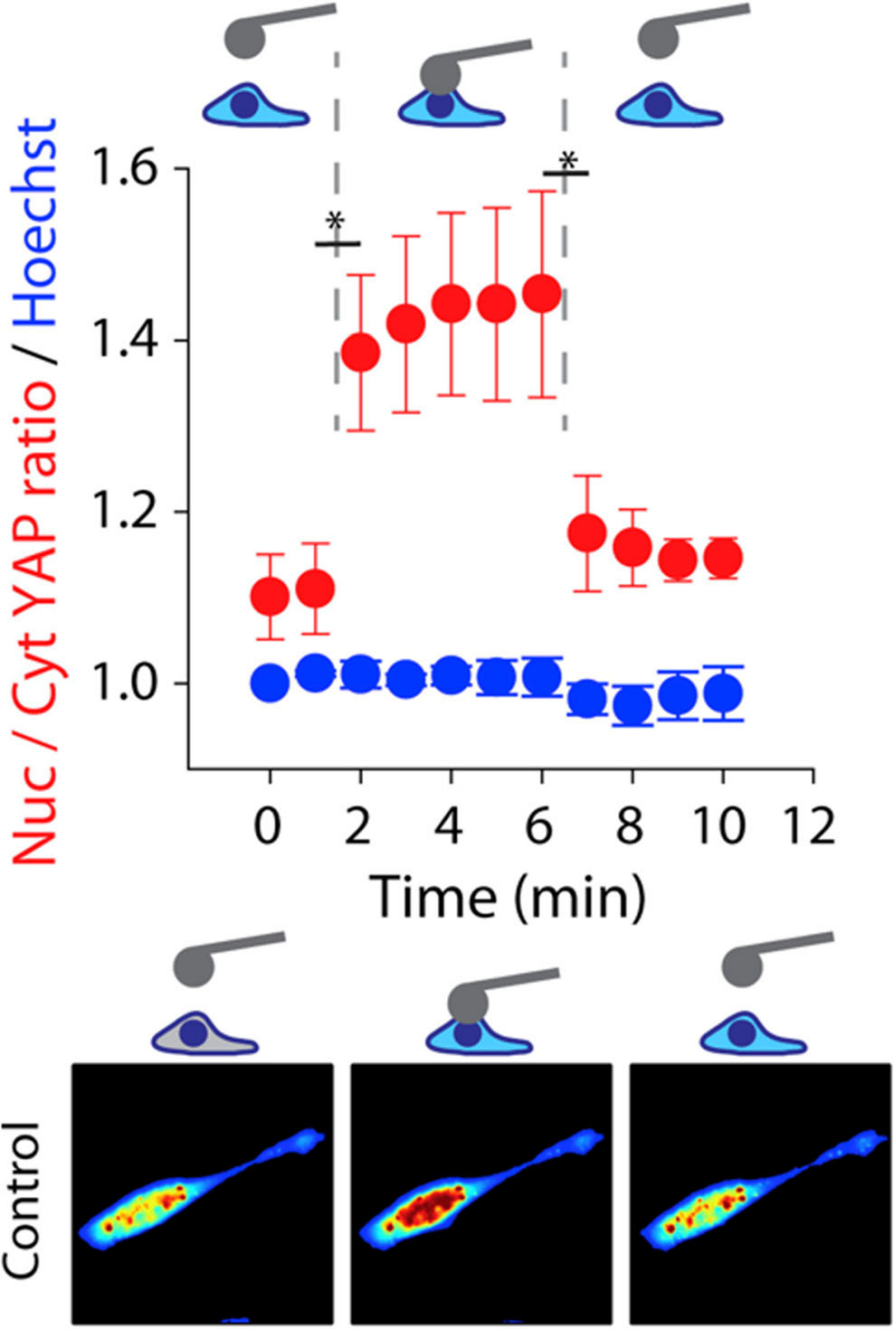
Top: Nuclear/cytosolic YAP ratio (red) and Hoechst nuclear average intensity (blue) for fibroblast cells seeded on 5 kPa gels (*n* = 9 cells) and transfected with EGFP-YAP during AFM indentation. Force was applied with an AFM cantilever with a 20-mm-diameter spherical tip. Sequentially: No force (1 min), 1.5 nN force (5 min), and no force (4 min). Bottom: Examples of color maps showing YAP fluorescence intensity in the conditions measured. Image taken from (Elosegui-Artola 2018) with permission

The question arisen is whether the flow of transcription factors in the nucleus is really due to a mechanical stimulus or if it was triggered by a molecular process. In fact, since scaffolds generate gradual and constant mechanical conditioning, it is reasonable to think that the mechanoregulation events could involve also not-immediate mechanical phenomena, such as chemical regulation given by importins and RAN-GTP concentration. However, the nuclear protein import results obtained from Elosegui-Artola by coupling the fluorescence microscopy atomic force microscopy (AFM) techniques, removed any doubt. They used AFM to apply a constant force to the cell nucleus and observed that force application increased the nuclear/cytosolic YAP ratio and that the YAP localization returned to the cytosol upon force release (Fig. 7). Therefore, they demonstrated that force application to the nucleus is enough to drive an immediate YAP nuclear translocation independently of RAN-GTP concentration and scaffold stiffness.

Despite MSCs being extremely sensitive to mechanical stimuli and therefore an optimal candidate to investigate mechanotransduction mechanisms, to the best of our knowledge, in literature, there are no other articles investigating the TF nucleocytoplasmic transport in MSCs. This is probably due to the difficulty of achieving MSCs expressing fluorescent molecules. In fact, standard DNA transfection procedures result less efficient in the case of stem cells (Maurisse 2010; Hamann 2019). On the other hand, other techniques useful to directly insert fluorescent probes into cells—like microinjection—are very time consuming and often unsuitable for application on non-standard substrates, such as truly 3D scaffolds like the Nichoid.

Nowadays, as well as understanding the effective mechanisms of the nuclear pore opening, the other important questions still open in the field of nucleocytoplasmic translocation are related to the characterization of TF-facilitated transport in relation to the cell environment and the nuclear shape.

Currently, we are studying the facilitated transport of a transcription factor involved in MSC differentiation towards the myocardial phenotype: MyoD (Vandromme et al. 1995). The main difference compared with the work conducted by Elosegui-Artola et al. is that the scaffold we use for cell growth (the Nichoid) is truly 3D and more representative of the physiological stem cell niche than a two-dimensional system.

**Fig. 8:**
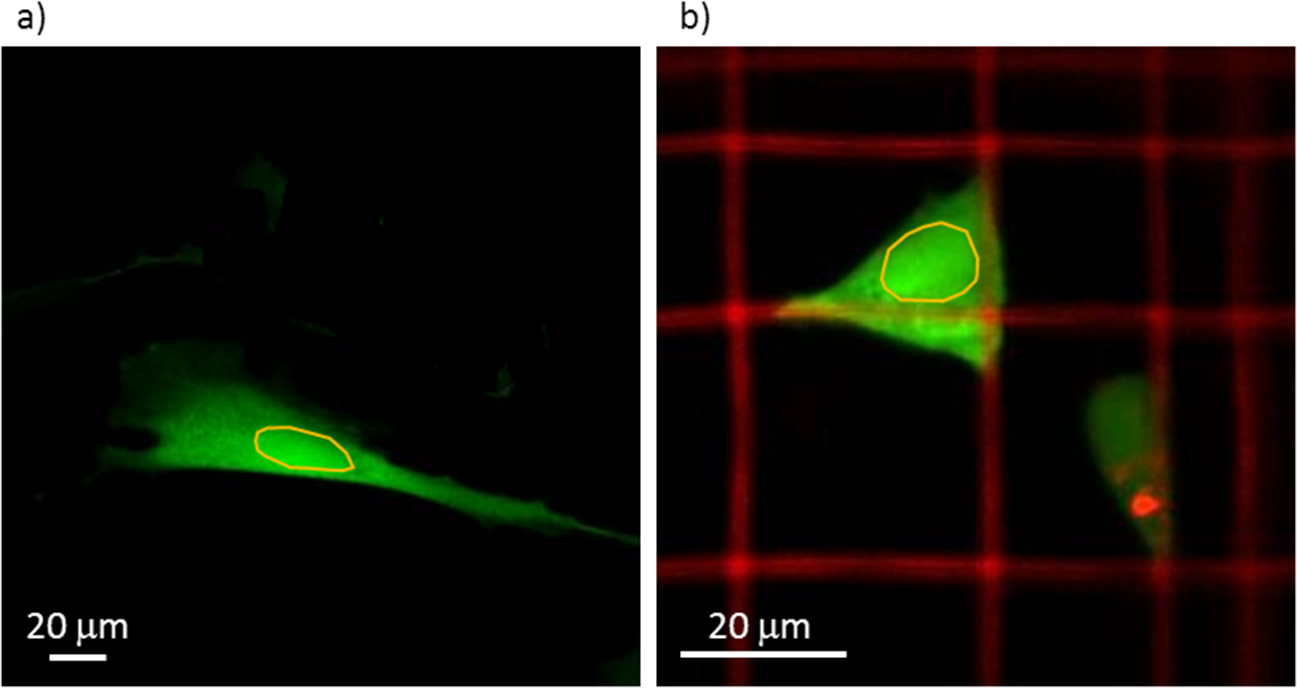
Images showing MSCs expressing MyoD-GFP grown on glass flat substrate (**a**) and in the Nichoid (**b**). In yellow, the cell nuclei are highlighted

We are considering two methods of investigation, FRAP and SMT, and facing their technical challenges. Apart from the difficulty of achieving MSCs expressing fluorescent transcription factors, each of these two methods has specific complexities. In the case of FRAP, for example, the extensive cellular three-dimensionality, induced by the cell growth in the Nichoid, complicates the measurement. In fact, what occurs during the bleaching phase of a nuclear ROI is that also many fluorescent proteins in the cytosol are bleached out. Moreover, as shown in Figs. 6 and 8, the nuclear section area on which the bleaching is carried out is about 3–4 times greater for cells grown on glass flat substrate than in the Nichoid. Although it is very difficult to carry out these measures, we have obtained the first results and we have developed a computational model of nuclear diffusion/deformation to better interpret the results from FRAP measurements.

**Fig. 9:**
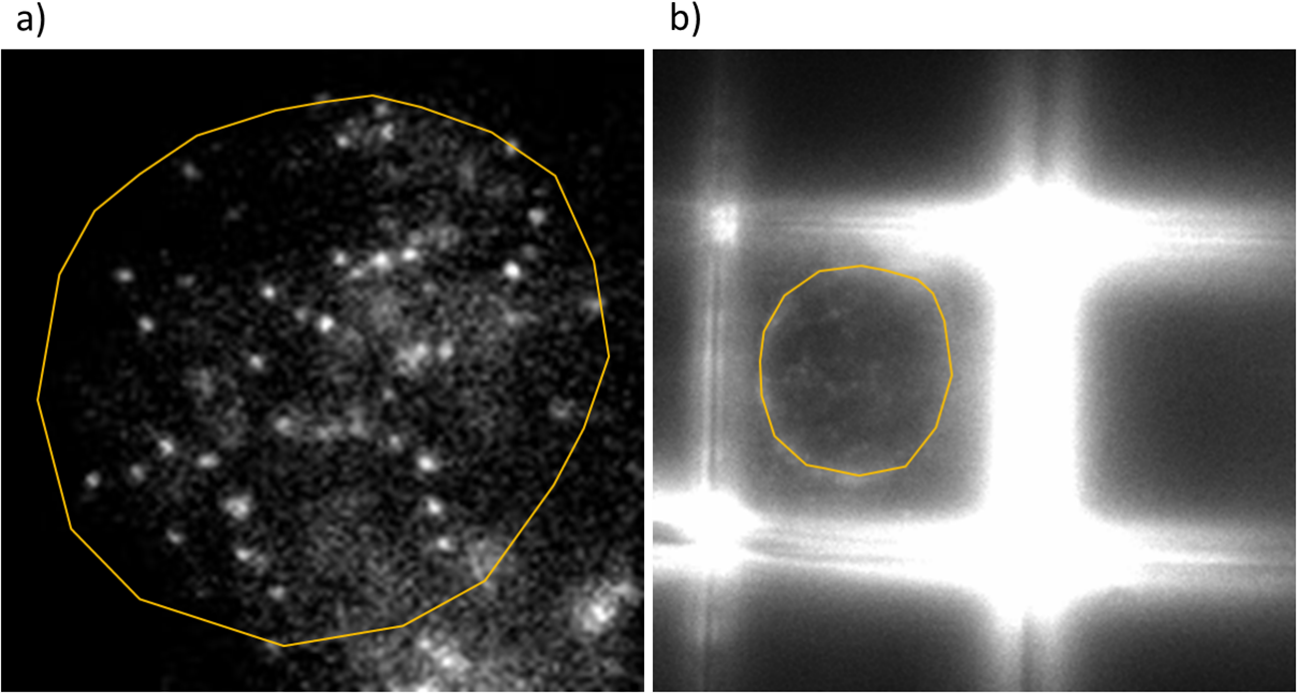
Examples of a SMT acquisition with HILO microscope on glass flat substrate (**a**) and in the Nichoid (**b**). Cells are MCF7 expressing fluorescent p53 protein. Images acquired at Istituto Scientifico Ospedale San Raffaele, Centro di Imaging Sperimentale, Milano

Regarding SMT, the major problems with this technique concern the three-dimensionality of the investigated systems and the sample fluorescence of the Nichoid. In fact, using a HILO microscope, the highly inclined beam does not allow the observation of the entire sample. Acquiring only a small part of the sample, the three-dimensionality is partially lost. This limitation could theoretically be overcome using a light sheet microscope. Instead, the Nichoid fluorescence problem is more complicated to solve. In fact, as explained in the previous paragraph, to make good SMT measurements, it is very important to maximize the SNR to contrast the brightness of the single molecules with respect to the background and to be able to make an accurate tracking. Since our sample fluoresces in the same wavelengths as the fluorophore, the application of this technique on cells grown in the Nichoid is still challenging (Fig. 9). We are trying to overcome this concerning aspect by varying the composition of the material in which it is produced and diminishing the Nichoid fluorescence.

## Conclusion

Mechanosensors and mechanotransduction mechanisms have a key role in modulating MSC fate by controlling the master switch between stemness maintenance and differentiation in these cells. As it has clearly emerged in some recent studies, the NPC plays a determinant role as a mechanosensor by regulating the nuclear import of transcription factors likely based on a stretch-activation mechanism. Despite the huge advancements in fluorescence microscopy to measure nucleocytoplasmic shuttling and to deepen the involvement of NPC in mechanotransduction, the optimal acquisition method is still to be defined, mainly due to the difficulty of transferring the existing techniques to 3D cell models.

The understanding of these mechanisms will allow the development and design of more performing substrates to mechanoguide and control cellular fate. These innovative systems could be used to improve cell-based therapy in regenerative medicine and in the field of personalized medicine.
